# Applying the multi-threat framework of stereotype threat in the context of digital gaming

**DOI:** 10.1371/journal.pone.0192137

**Published:** 2018-02-14

**Authors:** Charlotte R. Pennington, Linda K. Kaye, Joseph J. McCann

**Affiliations:** 1 Department of Health and Social Sciences, University of the West of England, Frenchay Campus, Coldharbour Lane, Bristol, United Kingdom; 2 Department of Psychology, Edge Hill University, Ormskirk, Lancashire, United Kingdom; Universitatsklinikum Tubingen, GERMANY

## Abstract

Females often report experiencing stigmatisation pertaining to their competency in digital gaming communities. Employing the principles of the multi-threat framework of stereotype threat, the current research examined the impact of gender-related stereotypes on females’ gaming performance and related self-perceptions. In Experiment 1, 90 females were assigned to one of three conditions in which they were primed that their performance would be either diagnostic of their personal (self-as-target) or gender group’s ability (group-as-target) or would be non-diagnostic of gaming ability (control). In Experiment 2, 90 females were primed that their performance would be judged by a group of other females (in-group source) or males (out-group source), or would be non-diagnostic of ability (control). Participants then completed a casual gaming task, as well as measures of competence beliefs, self-efficacy and self-esteem. Findings from Experiment 1 indicate that neither a self-as-target nor a group-as-target stereotype affected significantly gaming performance, or game-related self-efficacy, self-esteem and competency beliefs. Findings from Experiment 2 reveal further that females’ gaming performance and associated self-perceptions were not impacted significantly by an in-group or out-group source of stereotype threat. The discussion turns to potential explanations for these findings, proposing that females may not perceive negative gender-gaming stereotypes to be an accurate representation of their personal or social group’s gaming ability. We also discuss the implications of the experimental design and difficulty, as well as the potential for domain identification to moderate performance outcomes under stereotype threat.

## Introduction

Females frequently experience stigmatisation and are subject to discrimination within digital gaming communities [1]. Indeed, sexism towards females is a highly pervasive issue in digital gaming contexts [2] and portrayal of female characters in games themselves are typically hyper-sexualised and objectified [3]. Moreover, many females report that their gaming competency is often questioned and, consequently, feel marginalised by their male counterparts [4, 5]. For example, empirical evidence highlights that when gamers perceive another player to be female, rather than male, they are three times more likely to be verbally abusive [6]. It is therefore pertinent to consider the extent to which these stigmatised views impact upon the experiences of female players in digital gaming contexts.

Gender-based stereotyping in digital gaming is by no means a new phenomenon [7, 8], however what is less established are the psycho-social impacts for such stigmatised groups in these environments. That is, to what extent do stereotypical perceptions pertaining to females’ competence in gaming affect their performance and attitudes relating to the gaming domain? The current research sets out to address these questions by examining the influence of stereotype threat on females’ gaming performance, domain-specific competence beliefs, self-efficacy and self-esteem. In particular, we apply the principles of the multi-threat framework [9, 10] to assess its utility in understanding the potential detrimental effects of negative stereotypes for stigmatised females in gaming. Understanding these issues remains practically important given the under-representation of females in these contexts, as well as STEM (Science, Technology, Engineering and Mathematics) domains more generally [11]. Therefore, exploring the impacts of gender-related stereotypes in such a context presents an area of practical importance.

### Stereotype threat

Substantial attention has been paid to the effects of stereotype threat on performance in a range of domains, including academic tests [12–14] and athletic tasks [15]. The seminal work of Steele and Aronson [14] provided an understanding of how situational cues (i.e. the presentation of a salient negative stereotype) may hinder an individual’s ability on a stereotype-relevant task. The majority of work in this area demonstrates that females’ mathematical performance is substantially lowered when a mathematics test is framed as diagnostic of gender differences [13, 16]. Such findings pertaining to performance detriments appear to be robust [12, 17, 18], however, see [19] for a critical review. Despite this, the theory of stereotype threat has not been readily applied to other stigmatised populations, such as females within digital gaming contexts. Although some initial evidence has started to emerge in this regard [20, 21] there is still scant understanding of the impact that negative societal stereotypes may have on females’ gaming performance and associated attitudes. Specifically, there is some evidence that females underperform at gaming tasks under stereotype threat [21], although other findings are less clear-cut on this issue, and instead suggest that female players’ gamer identity is more prudent as a factor when exploring performance detriments associated with stereotype threat [20]. Nevertheless, no research to date has explored how variations in the form of stereotype threat (i.e., self-relevant or group-relevant) may impact upon females’ gaming performance.

### Distinguishing between distinct stereotype threats

The multi-threat framework proposes that individuals may experience distinct forms of stereotype threat, and that these may operate through numerous pathways, such as the *target* or the *source* of threat [9, 10]. The *‘target****’*** of stereotype threat refers to whether performance implications are attributable to the individual (i.e. self-as-target) or their social group (i.e. group-as-target). Specifically, females may encounter self-as-target stereotype threat when they perceive that a performance-evaluative task is self-characteristic of their personal ability. Conversely, they may encounter group-as-target threat when they perceive their performance to confirm, or reinforce, a negative stereotype about their social group [9, 16]. Moreover, the multi-threat framework posits that different factors may moderate the impact of these distinct stereotype threats on performance [9, 22, 23]. For example, Wout et al. [23] found that females in the domain of mathematics were more susceptible to group-as-target stereotype threat when they identified strongly with their gender, compared to those with lower gender identification. However, females with both high and low gender identification performed worse under self-as-target stereotype threat because it concerned their personal ability. Therefore, ‘self-as-target’ may be experienced distinctly relative to ‘group-as-target’ stereotype threats, and have a varied impact on performance outcomes.

In a similar vein, the *‘source’* of stereotype threat refers to an individual’s perception of *who* might judge their performance. Consistent with the underpinnings of Social Identity Theory [24–26], such priming techniques are designed typically to evoke in-group and out-group categorisation. Females may experience in-group threat when they believe that other members of their own social group will evaluate their performance (e.g., her gaming performance will be evaluated by other females). Yet they may face out-group stereotype threat when they believe that out-group members will judge performance (e.g., males) [9, 10]. Support for such distinction comes from research demonstrating that females underperform to a greater extent when they are required to complete a problem-solving task in the presence of other males relative to other females [27, 28]. As such, it could be expected that a strong sense of social identity to one’s stigmatised group (e.g., gender) may exert more pronounced effects on task performance under out-group relative to in-group stereotype threat conditions.

### Research overview

Across two experiments, the current research explored the principles set forth by the multi-threat framework [9] in the context of digital gaming. Experiment 1 manipulated the ‘target’ of stereotype threat by priming females with a self- or group-relevant stereotype about their gaming ability. Here it was predicted that females assigned to the group-as-target condition would score less points in a digital game relative to those in a non-threat control condition due to concerns that their performance may confirm the negative stereotype to be true of their gender group (a highly salient and stigmatised characteristic in gaming communities). Moreover, we expected that they would also underperform when primed with self-as-target stereotype threat due to concerns that they would confirm the negative societal stereotype as a true representation of their own ability [9]. We explored further whether gender identity moderated the stereotype threat-performance relationship, based on prior research suggesting that those who are highly gender identified may be more susceptible to group-as-target stereotype threat [21, 23]. Extending this, Experiment 2 manipulated the ‘source’ of the stereotype by priming females to believe that other in-group (i.e., females) or out-group members (males) would judge their performance. It was predicted that in-group stereotype threat would have a detrimental impact on females’ gaming performance (relative to a control) because of apprehensions that performance would confirm that their own group is legitimately devalued [9]. It was also predicted that females under out-group stereotype threat might underperform to a greater extent because such manipulation heightens the distinction between in-group and out-group members to evoke performance pressure [27, 28].

In addition to assessing gameplay performance, both studies explored the impact of these distinct forms of stereotype threat on additional psychological attitudes; namely, gameplay competence beliefs, self-esteem and gameplay self-efficacy. Such factors have not yet received empirical attention within the literature on stereotype threat and gaming, thus informing their inclusion in the current studies. It was predicted that stereotype threat would exert a detrimental impact on self-esteem, given research which suggests that self-esteem is affected negatively in instances of stigmatisation [29], particularly when an upward social comparison is primed [30]. Similarly, equivalent effects were expected for one’s perceptions of domain-specific self-efficacy and competence beliefs [31–34].

## Experiment 1 –Target of stereotype threat

### Participants

Ninety female participants (*M*age = 19.39, *SD* = 2.88) participated in return for course credit or monetary remuneration. As the intention was to study these issues in a casual gaming context, it was not a priority to recruit those who specifically identified as “gamers”, particularly given that female players tend not to identify as such [35–37]. Of this sample, 17.8% identified themselves as being a “gamer” (primarily “casual” or “social”), with 8.9% of this sub-sample reporting that they played for an average of 1–5 hours per week, 6.7% for less than 1 hour, and 7.8% between 6–20 hours per week. Prior to participating in any tasks, they were equally and randomly allocated to one of three experimental conditions; 1), self-as-target stereotype threat; 2), group-as-target threat; or 3), a non-threat control. Sample size was determined prior to commencing with the study based on logistical and monetary considerations, and data collection stopped once 30 participants had been recruited for each experimental condition.

### Stereotype threat manipulations

In line with the multi-threat framework [9, 10], participants were primed with either a self-as-target or group-as-target stereotype. In order to evoke stereotype threat effectively, prior research suggests that participants should be knowledgeable of the negative societal stereotype in order for it to impact performance [9]. Accordingly both the self-as-target and group-as-target primes included reference to the negative stereotype pertaining to perceived gender differences in gameplay performance, but differentiated whether participants were told that their performance would be indicative of their personal gaming aptitude (self-as-target) or reflective of their social group’s alleged proficiency (group-as-target). Participants in the “self-as-target” condition were primed with the following written information designed to heighten the salience of their personal identity:

“In today’s session, we want to assess gameplay ability by asking you to play the following game. Typically, males have been shown to outperform females on this specific game. Your performance on this will therefore be used to help us establish your **personal gameplay ability**”.

Participants in the “group-as-target” condition were primed with the following information, which was developed to heighten the salience of their social identity:

“In today’s session, we want to assess gameplay ability for male and females by asking you to play the following game. Typically, males have been shown to outperform females on this specific game. Your performance on this will therefore be used to help us establish **females’ gameplay ability**.”

Participants in the control condition were not primed with any gender-related stereotype and instead received the following written information, designed to nullify stereotype threat effects [38].

“In today’s session, we want to assess various factors relating to gameplay. This task is **not diagnostic of ability**”.

### Measures

#### Social identity

Research suggests that individuals are more susceptible to stereotype threat when they identify with their social group [23, 39]. As such, before receiving any of the experimental manipulations, participants completed the *three-dimensional strength of group identification scale* [40] to measure their identification with their gender group. They answered 12 questions such as “I often think about being a female”, and “I feel strong ties to other females” on 7-point Likert scale (1 = Strongly Disagree, 7 = Strongly Agree; Cronbach’s *a* = .74). Participants’ mean social identity score was 59.64 (*SD* = 8.59, range 38–80), suggesting a moderate-to-high level of gender identification [41]. Furthermore, social identity did not differ as a function of experimental condition (*F*(2, 86) = 1.38, *p* > .05, partial eta squared = .031) suggesting that this factor was adequately controlled for.

#### Gameplay performance

Participants were required to play “Red Ball 4”, an online game which was played through a PC platform. In this slide-and-scroller game, participants operated a ball through a maze with a series of obstacles to accumulate points. The amount of points gained after five minutes was taken as an overall measure of performance. This game was chosen because we wanted a game that was not perceived as being too masculine or feminine, and instead would be relatively neutral in nature. This was to ensure that the game itself was not an experimental confound in priming any gender-related performance outcomes. As an assurance of this, participants rated how masculine/feminine they perceived the game to be to ensure that the game design did not inadvertently evoke stereotype threat (1 = feminine, 5 = neutral/neither, 9 = masculine). Overall, the game was rated neutrally (*M* = 4.92, *SD* = .89), and such ratings did not differ as a function of experimental condition *F*(2, 85) = .60, *p* = .551, partial eta squared = .01.

#### Gameplay competence beliefs

Participants answered two questions to assess their perceived gaming competence (e.g., “To what extent do you think that you were competent in the gaming task?”) and the importance of their gaming competence (e.g., “To what extent was it important to you to be competent in the gaming task?”) on a 9-point Likert scale (1 = Not at all, 9 = Extremely). Mean responses to these two questions were analysed separately. These questions therefore provided a measurement of perceived *task importance* and *task competence*.

#### Self-esteem

The *Rosenberg Self-Esteem Scale* [42] was implemented as a measure of self-esteem. Participants responded to 10 questions, such as “I take a positive attitude towards myself”, on a 4-point scale (1 = Strongly Disagree, 4 = Strongly Agree; Cronbach’s *a* = .83). A total score of self-esteem was computed.

#### Self-efficacy

An adapted version of the self-efficacy scale [43] was used to measure self-efficacy in gaming. Participants endorsed their agreement to 10 questions, such as “I could solve most problems in gameplay if I was to invest the necessary effort” on a 4-point scale (1 = Not at all true, 4 = Exactly true; Cronbach’s *α* = .84). A total score of self-efficacy was computed.

#### Stereotype threat manipulation check

Two questions were included to measure the effectiveness of the stereotype threat primes. Participants were asked: “Do you know of a negative stereotype regarding females being less competent as gamers compared to males?” (Yes or No), and “To what extent do you believe that males would be more competent gamers than females at the game you have just played?” (1 = Females better, 5 = Neutral/no difference, 9 = Males better).

### Procedure

**Fig 1:**
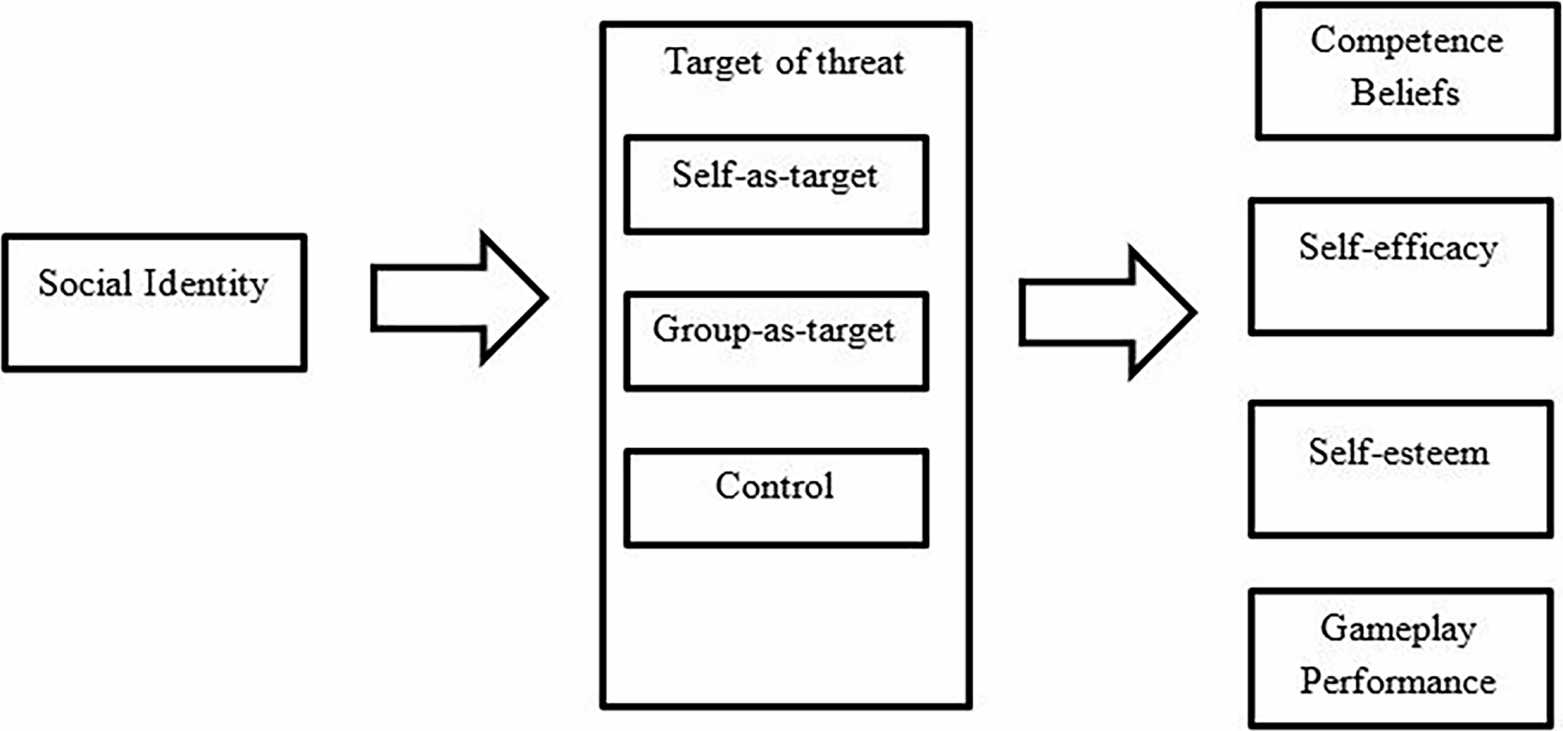
Overview of the experimental procedure for Experiment 1.

The Research Ethics Committee within the Department of Psychology deemed this research ethically sound and approved the protocol. All research participants provided informed written consent prior to taking part and declared that they understood fully the requirements of the study and their ethical rights. Participants then completed a demographics questionnaire and a measure of social identity, before being provided with written information that corresponded to their assigned experimental condition. They then completed the gaming task (Red Ball 4) for five-minutes. This gameplay period was timed by the researcher, using a stopwatch, with participants’ score recorded at the end of the session. Following this, participants completed a battery of questionnaires to assess their gameplay competence beliefs, gaming self-efficacy [43] and self-esteem [42]. These were completed in this order for all participants, across all experimental conditions. Finally, they responded to two manipulation checks before being fully debriefed regarding the true purpose of the experiment. See Fig 1 for schematic overview of the procedure.

### Results

#### Analytic Strategy

A series of one-way Analysis of Variance (ANOVA) tests were conducted to examine the impact of stereotype threat on gaming performance, competence beliefs, self-esteem and self-efficacy. Simple main effects were elucidated using a Bonferroni-correction and effect sizes are reported in Cohen’s *d* indices [44]. Positive effect sizes indicate that the findings were in line with experimental predictions, whereas negative effect sizes indicate that the findings were contrary to predictions. Social identity was examined as a moderator of self- and group-as-target stereotype threat effects using path analysis (PROCESS macro [45]). This was conducted to elucidate whether gameplay performance decrements may vary based on participants’ social identity (i.e., the extent to which they identify with being a female gamer).

#### Gaming performance

One participant’s gameplay score was excluded from the analyses because they represented an extreme outlier (identified using boxplots). There was no significant main effect of experimental condition on gameplay performance, *F*(2,86) = 1.85, *p* = .163, partial eta squared = .041. Simple main effects indicated that participants assigned to the self-as-target (*M* = 3316.67, *SD* = 1916.37) and group-as-target stereotype threat conditions (*M* = 2627.59, *SD* = 1054.55) performed similarly to those in the control condition (*M =* 3233.33, *SD* = 1378.74), both *p* > .37, *d* = .05 and *d* = −.49, respectively. No significant difference was found between the self-as-target and group-as-target conditions, *p* = .24, *d* = − .45. In contrast to predictions, social identity (i.e., gender identification) was not found to moderate the relationship between self- or group-as-target stereotype threat and gaming performance, *p* > .52.

#### Gameplay competence beliefs

There was no significant main effect of experimental condition on perceptions of task competence, *F*(2,86) = 1.32, *p* = .272, partial eta squared = .03. Participants in both the self-as-target (*M* = 4.73, *SD* = 1.62) and group-as-target conditions (*M* = 4.10, *SD* = 1.86) reported similar evaluations of gaming competence compared to the control condition (*M* = 4.69, *SD* = 1.54), *p* > .54, *d* = − .03 and *d* = .34, respectively. No significant difference was found between the self-as-target and group-as-target conditions, *p* = 1.00, *d* = − .36. There was also no significant main effect of experimental condition on perceptions of task importance, *F*(2,86) = .260, *p* = .772, partial eta squared = .006, with participants in the self-as-target (*M* = 5.20, *SD* = 2.44) and group-as-target conditions (*M* = 5.30, *SD* = 2.17) reporting similar evaluations of task importance compared to the control (*M* = 5.59, *SD* = 1.70), *p* = 1.00, *d* = .19 and *d* = .15, respectively. No significant difference was found between the self-as-target and group-as-target conditions, *p* = 1.00, *d* = .04.

#### Self-esteem

There was no significant main effect of experimental condition on self-esteem, *F*(2, 87) = 1.53, *p* = .222, partial eta squared = .034. Self-reported self-esteem did not differ significantly between the self-as-target (*M* = 29.97, *SD* = 3.86) and group-as-target conditions (*M* = 29.00, *SD* = 3.71) relative to the control condition (*M* = 28.17, *SD* = 4.36), *p* > .25, *d* = −.44 and *d* = −.21, respectively. There was no significant difference in levels of self-esteem between the self-as-target and group-as-target conditions, *p* = 1.00, *d* = —.27.

#### Self-efficacy

There was no significant main effect of experimental condition on game-specific self-efficacy, *F*(2,87) = .601, *p* = .550, partial eta squared = .014. Participants’ self-reported self-efficacy did not differ significantly between the self-as-target (*M* = 27.53, *SD* = 4.10) and group-as-target conditions (*M* = 26.60, *SD* = 3.89) relative to the control condition (*M* = 27.63, *SD* = 4.08), *p* > .97, *d* = .02 and *d* = .26, respectively. There was no significant difference in self-efficacy between the self-as-target and group-as-target conditions, *p* = 1.00, *d* = −.23.

#### Stereotype threat manipulation check

Across all conditions, 85% of participants reporting knowing about the negative gender stereotype pertaining to females’ gaming competence. Exploratory analyses indicate that the results do not change when excluding those who reported not knowing about the negative gender–gaming stereotype were excluded from the stereotype threat conditions. There was no significant main effect of experimental condition on the second manipulation check, *F*(2,87) = .125, *p* = .883, partial eta squared = .003. Participants in both the self-as-target (*M* = 5.60, *SD* = 1.52) and group-as-target conditions (*M* = 5.70, *SD* = 1.12) believed females and males to be similarly competent gamers and this did not differ significantly compared to the control condition (*M* = 5.53, *SD* = 1.22), *p* = 1.00, *d* = − .15 and *d* = − .05 respectively. See Table 1 for descriptive statistics.

**Table 1:** Descriptive statistics of variables by experimental condition.

	Experimental Condition		
	Self-as-target	Group-as-target Mean (SD)	Control
**Gameplay performance**	3316.67 (1916.37)	2627.59 (1054.55)	3233.33 (1378.74)
Social identity*	58.83 (9.04)	61.73 (8.40)	58.33 (8.22)
**Task Competence**	4.73 (1.62)	4.10 (1.86)	4.69 (1.54)
**Task Importance**	5.20 (2.44)	5.30 (2.17)	5.59 (1.70)
**Self-esteem**	29.97 (3.86)	29.00 (3.71)	28.17 (4.36)
**Self-efficacy**	27.53 (4.10)	26.60 (3.89)	27.63 (4.08)
**Manipulation Check**	5.60 (1.52)	5.70 (1.12)	5.53 (1.22)

*Note: Social identity was measured before the experimental primes but is denoted here for reference.

### Discussion

Underpinned by the multi-threat framework [9, 10], Experiment 1 examined the impact of self- and group-relevant stereotype threat on females’ gaming performance, as well as a number of theoretically relevant factors pertaining to self-perceptions, such as competence beliefs, self-esteem, and self-efficacy. In contrary to our experimental predictions, the current findings reveal little empirical support for the notion of stereotype threat operating within this casual digital gaming context. Specifically, the salience of a self- or group-relevant stereotype did not appear to influence significantly gameplay performance, nor did it impact adversely on competence beliefs, self-esteem, or gameplay self-efficacy. Experiment 2 set out to examine a second facet of the multi-threat framework: the source of stereotype threat. Specifically, we aimed to establish whether a distinction between in-group and out-group categorisation might impact gaming performance.

## Experiment 2

### Participants

Ninety female participants (*Mage* = 24.29, *SD* = 9.71) were allocated equally and randomly to one of three stereotype conditions (*n* = 30 in each): 1), in-group stereotype threat; 2), out-group stereotype threat; and 3), a non-threat control. Of the sample, 18.89% identified themselves as being a “gamer” (either “casual” or “social”), with a sub-sample of 52.9% reporting that they played for an average of 1–5 hours per week, 35.5% between 6–20 hours and 11.8% for less than 1 hour per week.

### Stereotype threat manipulations

Participants assigned to the in-group stereotype threat condition were led to believe that their performance would be viewed by another group of female students. This manipulation was devised to influence participants to perceive that their performance may confirm the negative stereotype in the minds of in-group others (c.f., “group reputation threat in-group” [9], pp. 113). They were primed with the following written information:

“In today’s session, we want to assess gameplay ability by asking you to play the following game. Typically, males have been shown to outperform females on this specific game. Your performance on this game will **viewed by a group of female students** as part of another project.”

Participants in the out-group stereotype threat condition were led to believe that their performance would be viewed by a group of male students, and thus reinforce the stereotype in the minds of out-group members (c.f., “group reputation threat out-group” [9]). They were primed with the following:

“In today’s session, we want to assess gameplay ability by asking you to play the following game. Typically, males have been shown to outperform females on this specific game. Your performance on this game will be **viewed by a group of male students** as part of another project.”

Participants in the control condition were provided with the same information as Experiment 1.

### Measures and procedure

**Fig 2:**
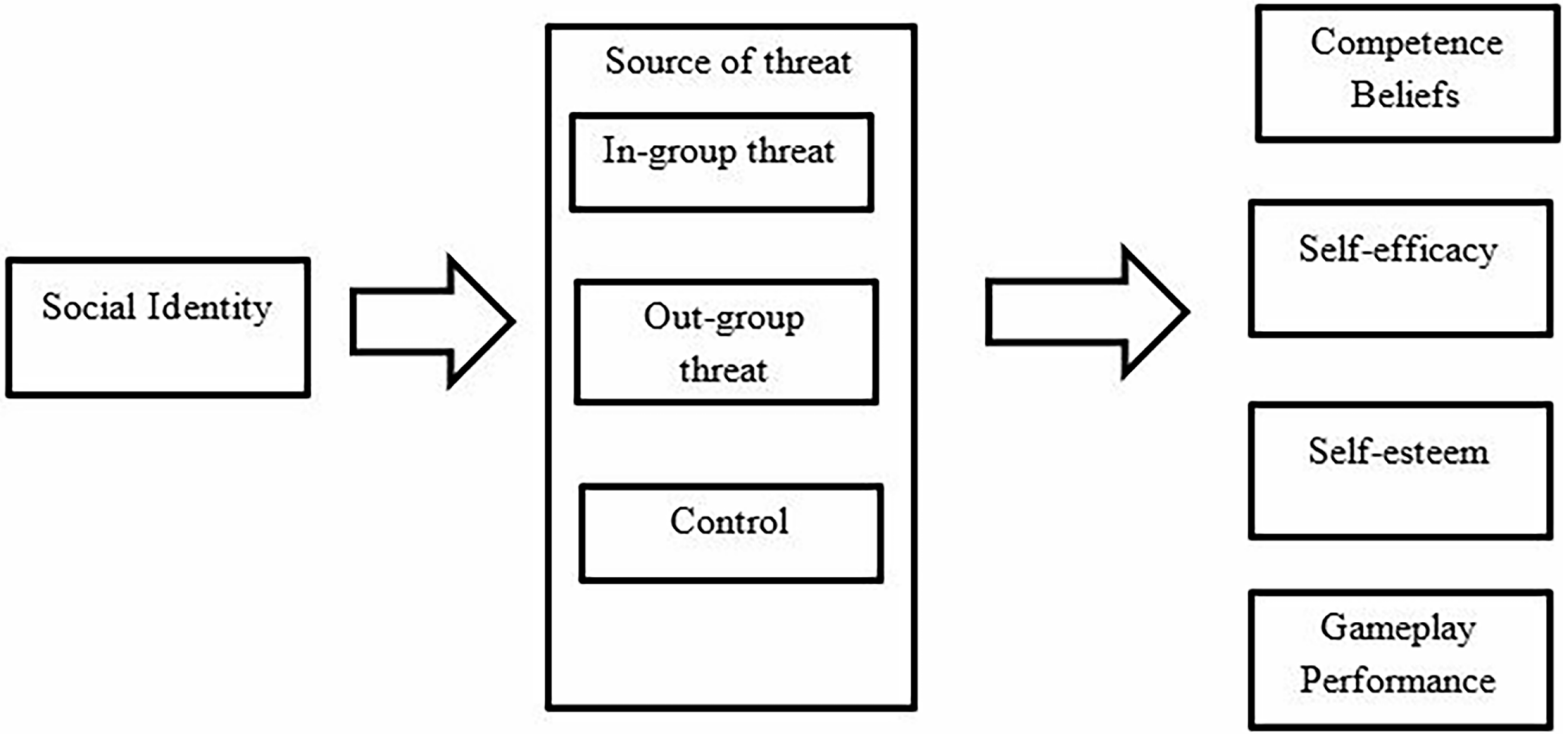
Overview of the experimental procedure in Experiment 2.

Experiment 2 followed the same ethical assurances, materials and procedures as employed in Experiment 1. See Fig 2 for procedure overview. To note, we again obtained data on how masculine/feminine participants perceived the game to be. Similarly to Experiment 1, the game was perceived as gender-neutral (*M* = 4.86, *SD* = .87), and these ratings did not differ significantly as a function of experimental condition *F*(2, 87) = .1.35, *p* = .264, partial eta squared = .03.

### Results

#### Gaming performance

Two participants’ outlying gameplay scores were excluded from the analyses (identified using boxplots). There was no significant main effect of experimental condition on gameplay performance, *F*(2, 85) = .839, *p* = .436, partial eta squared = .019. Contrary to predictions, participants assigned to the in-group (*M* = 2706.67, *SD* = 1285.98) and out-group threat conditions (*M* = 2382.14, *SD* = 1487.99) performed similarly to those in the control condition (*M =* 2276.67, *SD* = 1237.82), *p* > .65, *d* = −.34, and *d* = −.08, respectively. There was no significant difference in gameplay performance between the in-group and out-group threat conditions, *p* = 1.00, *d* = .23. Social identity was not found to moderate the relationship between self- or group-as-target stereotype threat on gaming performance, *p* > .21.

#### Gameplay competence beliefs

There was no significant main effect of experimental condition on perceptions of task competence, *F*(2, 87) = 1.91, *p* = .154, partial eta squared = .042. Participants in both the in-group (*M* = 4.00, *SD* = 1.39) and out-group (*M* = 3.87, *SD* = 1.83) conditions perceived themselves to be similarly competent compared to those in the control condition (*M* = 4.73, *SD* = 2.22), *p* > .22, *d* = .39 and *d =* .42, respectively. No significant differences were found between the in-group and out-group threat conditions, *p* = 1.00, *d* = − .08. There was also no significant main effect of experimental condition on perceptions of task importance, *F*(2, 84) = 2.06, *p* = .134, partial eta squared = .047. Participants in the in-group (*M* = 4.97, *SD* = 2.51) and out-group (*M* = 6.11, *SD* = 2.15) conditions rated their task competency to be similarly important compared to those in the control condition (*M* = 4.97, *SD* = 2.58), *p* > .24, *d* = 0.0 and *d* = −.48, respectively. No significant differences were found between the in-group and out-group threat conditions, *p* = .24, *d* = −.49.

#### Self-esteem

There was a significant main effect of experimental condition on self-esteem, *F*(2, 87) = 3.34, *p* = .040, partial eta squared = .07. Simple main effects indicated that participants in the out-group source stereotype threat condition (*M* = 27.47, *SD* = 4.31) reported marginally lower self-esteem compared to those in the in-group source condition (*M* = 30.00, *SD* = 3.43), *p* = .057, *d* = .65. There was no significant difference between the in-group (*M* = 30.00, *SD* = 3.43) and out-group conditions (*M* = 27.47, *SD* = 4.31) relative to the control condition (*M* = 27.83, *SD* = 4.50), *p* = .13, *d* = −.54 and *d* = .08, respectively.

#### Self-efficacy

There was no significant main effect of experimental condition on self-efficacy, *F*(2, 86) = 1.78, *p* = .175, partial eta squared = .040. Self-efficacy did not differ significantly between the in-group (*M* = 26.23, *SD* = 3.37) and out-group conditions (*M* = 25.70, *SD* = 4.65) relative to the control condition (*M* = 28.14, *SD* = 7.02), *p* > .23, *d* = .35 and *d* = .41, respectively. There was no significant difference in game-specific self-efficacy beliefs between the in-group and out-group conditions, *p* = 1.00, *d* = .13.

#### Manipulation check

Across all conditions, 80% of participants were knowledgeable about the negative gender stereotype pertaining to females’ gaming competence. As in Experiment 1, the reported findings do not change when those who were not knowledgeable of this stereotype are excluded only from the stereotype threat conditions. There was also no significant main effect of experimental condition on the second manipulation check, *F*(2, 87) = 1.54, *p* = .220, partial eta squared = .03. Specifically, participants in both the in-group (*M* = 5.93, *SD* = 1.23) and out-group threat conditions (*M* = 5.53, *SD* = 1.28) believed females and males to be equally competent gamers and this did not differ compared to the control condition (*M* = 5.43, *SD* = .97), *p* > .30, *d* = −.45 and *d* = −.08. See Table 2 for descriptive statistics.

**Table 2:** Descriptive statistics of experimental variables by experimental condition.

	Experimental Condition		
	In-group threat	Out-group threat Mean (SD)	Control
**Gameplay performance**	2706.67 (1285.98)	2382.14 (1487.99)	2276.67 (1237.82)
**Social identity**	60.47 (7.48)	56.48 (8.61)	60.17 (12.66)
**Task Competence**	4.00 (1.39)	3.87 (1.83)	4.73 (2.23)
**Task Importance**	4.97 (2.51)	6.11 (2.15)	4.97 (2.58)
**Self-efficacy**	26.23 (3.37)	25.70 (4.64)	28.14 (7.02)
**Self-esteem**	30.00 (3.43)	27.47 (4.31)	27.83 (4.50)
**Manipulation Check**	5.93 (1.23)	5.53 (1.28)	5.43 (.97)

### Discussion

Consistent with Experiment 1, the findings from Experiment 2 indicate that priming an in-group or out-group stereotype did not significantly lower females’ gaming performance. Moreover, these difference ‘sources’ of stereotype threat did not appear to affect domain-related competence beliefs, self-esteem or self-efficacy. The general discussion turns to factors that may explain these overall findings, with a focus on stereotype endorsement, task difficulty and domain identification.

## General discussion

Following the theoretical implications set forth by the multi-threat framework [9, 10], the current research examined the influence of distinct forms of stereotype threat on females’ gaming performance. Experiment 1 manipulated the ‘target’ of threat by priming females with a negative self-as-target or group-as-target stereotype. Experiment 2 manipulated the ‘source’ of threat by leading females to believe that their performance would be evaluated by in-group or out-group others. Findings from both Experiment 1 and 2 indicate little support for distinct forms of stereotype threat manifesting any detrimental outcomes on females’ performance or related attitudes in the domain of casual gaming. This is largely contrary to existing findings in the wider literature, which have shown stereotype threat to diminish performance on a variety of tasks and across distinct populations [16]. It also contrasts with prior theoretical and empirical work, which has suggested that distinct forms of stereotype threat (e.g., self-as-target and group-as-target) may have unique influences on performance [23], be moderated by different factors [9, 22], and require different interventions to overcome [22].

Such findings therefore reveal a positive indication that different stereotype primes may not have a detrimental impact upon females’ gameplay performance and related attitudes in the domain of casual gaming. Indeed, effect sizes obtained suggest that any effects–either positive or negative—were small, and many were in the opposite direction to our experimental predictions [44]. Even though females generally appear to be knowledgeable of gender-related gaming stereotypes, these findings may indicate that they do not perceive such stereotypes to be an accurate representation of their personal gaming aptitude (self-as-target), nor a true reflection of their social group’s ability (group-as-target). This suggestion is supported by the findings of the manipulation check within the current research, which indicated that although females reported being knowledgeable about negative gender-related stereotypes, they did not tend to endorse females’ gaming competence as significantly lower than males. Indeed, previous findings also indicate that females generally disagree with gender-related stereotypical beliefs [46], and even hold implicit attitudes towards females being more competent gamers than they do males [20]. With this in mind, it may be that females are aware of negative gaming-related gender stereotypes, but do not perceive them to be valid, or possibly engage in strategies to disconfirm the negative stereotype.

Presenting an alternative perspective, it is plausible that the majority of females may experience stereotype threat to some extent within digital gaming communities and, as such, stereotype threat primes may not have an additive adverse effect on performance in experimental settings [47]. In support of such suggestion, approximately 80% of our overall sample reported being aware of negative gender-related stereotypes in gaming, and this did not differ as a function of assigned experimental condition. Consequently, we cannot exclude the possibility that female participants in the control condition were experiencing stereotype threat, which may have masked any differential performance outcomes across experimental conditions. This highlights the pervasiveness of gender stereotypes in digital gaming arenas, and the stigma that is often reported by female players [2, 6]. It is perhaps therefore fruitful to consider ways of alleviating negative societal stereotypes pertaining to female gameplay, which may be realised through priming superordinate identity (gamer identity) rather than gender identity within gaming contexts. Indeed, recent research has demonstrated such strategy to have a positive impact on females' gaming performance [20].

Another explanation for the current findings may be that the task was not difficult enough to evoke situational performance pressure, with task difficulty documented as a moderator of the stereotype threat-performance relationship [48, 49]. Exploring whether females are more susceptible to the impact of stereotype threat within difficult gaming tasks is therefore an important area of further enquiry, particularly to establish whether all types of gaming tasks are immune to stereotype threat manipulations. Relatedly, it is conceivable that tasks which are more procedural in nature (e.g., gaming) may not be as vulnerable to stereotype threat compared to tasks which co-opt working memory processes [48], such as academic tests, which are highly prominent within this literature [12–14].

Putting the current findings into a real-world context, it would be interesting to examine whether stereotype threat effects occur in digital gaming environments, such as online or multi-player gaming. This is pertinent in light of a more recent issue of group-based contexts, which is becoming apparent in the stereotype threat literature [16, 27, 28]. Specifically, research shows that stereotype threat performance decrements are alleviated when testing takes place with other group members who share the target stigmatised characteristic [16]. The implications of this are key, particularly when considering the plethora of real-world gaming tasks that occur typically in groups, in which stereotype threat may operate. For example, research suggests that females may encounter harassment within online digital forums and multi-player domains [6], and it is conceivable that such experiences may shape females’ perceptions of, and participation in, gaming arenas. It would therefore be interesting to explore the role of gender composition within online gaming tasks, and whether this results in differential performance outcomes. As well as providing an assessment of stereotype threat effects *in-vivo*, such recommendations proffer advantages to lab-based experiments. For example, stereotype threat is evoked typically in the lab through the simple presentation of a gender-related stereotype at the start of the task. It is therefore possible that social priming effects are limited in their duration within experimental studies [50]. However, stereotype threat may be more salient throughout online gaming tasks, because females may face negative attitudes continuously throughout gameplay, and this may provide a more reliable assessment of the long-lasting impact of stereotypes on performance and related attitudes. Performance outcomes, particularly as a result of group composition, have also been found to manifest themselves differently dependent on the nature of gameplay in respect of cooperative versus competitive play [51], thus a more specific exploration into group processes underpinning stereotype threat in domain of digital gaming is an avenue for future research.

### Limitations and future research recommendations

Although we provide two experiments that test the multi-threat framework in a novel context, this research is not without limitations. A key limitation pertains to whether a casual gaming context is sufficient to elicit strong domain identification. That is, casual gaming may not necessitate one to identify as a “gamer” compared to games which may be characterised as more “hard-core” in nature [52]. Additionally, it has been found that marginalised groups in gaming, such as females, may not identify themselves as gamers [36, 37]. As such, there is a likelihood that participants may not have identified strongly with the domain of gaming and consequently were not susceptible to stereotype threat effects.

In a similar vein, we have focused on stereotype threat in a casual gaming context, which arguably is a female-dominated domain relative to other gaming domains [35]. We cannot yet establish whether these findings would differ in “hard-core” gaming contexts, which may be more stereotypically considered “male space” [11]. Therefore, researchers should be cautious in their claims of a “one-size-fits-all” approach to the existence and operationalisation of stereotype threat, without due attention to its nuances across tasks and domains.

It is also noteworthy to evaluate the validity of the different stereotype threat manipulations employed the current study, particularly since they are based on the theoretical premises put forth by the multi-threat framework [9, 10], and equivalent priming techniques that have proved to be effective in prior research [22, 23]. Specifically, it could be argued that the self-as-target and group-as-target primes are not distinct, as the multi-threat framework implies, because they both refer to gender differences in performance, thus evoking the salience of group membership. As a result, it is not known within the current study whether females apprehended that their gaming performance would be used as a diagnostic indicator of their personal aptitude (self-a-target), or to reinforce the negative stereotype as a true representation of their social group’s gaming proficiency (group-as-target) because the manipulation checks did not delineate between these two 'distinct' primes. Future research should overcome this by elucidating whether participants are accurately able to identify, and distinguish between, self-as-target and group-as-target stereotype threat manipulations [22]. Empirical support for the existence of distinct stereotype threats would bolster the theoretical underpinnings of the multi-threat framework [9, 10, 22], and provide a rationale for employing different self- and group-relevant priming techniques within future research. It might also shed light onto why research is yet to reach a consensus regarding the underling mechanisms of the stereotype threat-performance relationship [see 18], and the robustness of stereotype threat priming techniques across different populations and tasks.

In respect of the psychological outcomes of self-efficacy and competence beliefs, it is probable that participants were not able to make accurate assessments of these, with no source of comparison on which to base their personal gaming performance appraisal. Future research could therefore explore the influence of explicit performance feedback or relative comparisons (leader-boards) as a means to evoke stereotype threat [21]. This would welcome bespoke game development, in which feedback mechanisms are able to be manipulated and built-in to games to permit an exploration of how stereotype threat may influence gameplay performance and psychosocial attitudes within gaming contexts.

Finally, the measures of competence beliefs, self-esteem and self-efficacy were not counterbalanced between participants and it could therefore be argued that order effects were introduced into the experimental design. Although we found non-significant findings as a function of stereotype condition for all of these self-report questionnaires, it is advised that future studies counterbalance measures to ensure that, for example, females’ self-reported global self-esteem does not influence their reports of gameplay-specific self-efficacy beliefs [53].

## Conclusion

The current findings indicate that the salience of a negative self- or group-relevant stereotype (Experiment 1) did not appear to influence females’ gameplay performance or related attitudes. Furthermore, perceiving that in-group or out-group others may judge performance negatively did not evoke stereotype threat effects (Experiment 2). Whilst we acknowledge there may be explanations for these findings, such as task difficulty, stereotype endorsement, and the casual gaming context, we provide a positive indication that priming negative gender-related stereotypes does not *always* result in detrimental performance outcomes.

We advocate that there is still much to explore in respect of understanding what specific conditions may evoke experiences of stereotype threat in digital gaming communities, as well as an exploration of different gaming tasks that might elicit different effects. Specifically, we present findings in a casual gaming context, yet it is not understood whether these are applicable in other gaming domains, such as hardcore gaming that is viewed stereotypically as a more masculine relative to feminine pursuit. We also recommend future research to explore females’ gameplay experiences within real-world gaming context, particularly in light of the stigma and prejudice they face within such gaming communities [1–4]. Future work could also examine strategies to enable females to effectively deal with these stereotypical views, with a view to widening their participation in digital gaming communities, and STEM domains more generally.

## Supporting information

S1 File Study 1 data. (XLSX)

S2 File Study 2 data. (XLSX)

## References

[1] Jenson J, Fisher S, de Castell S. Disrupting the gender order: Leveling up and claiming space in an after-school video game club. Int J Gend Sci Technol. 2011; 3(1): 148–169.

[2] Fox J, Tang W. Sexism in online video games: The role of conformity to masculine norms and social dominance orientation. Comput Human Behav. 2014; 33: 314–320.

[3] Burgess MCR, Sterner SP, Burgess SR. Sex, Lies and Video Games: The portrayal of male and female characters in video game covers. Sex Roles. 2007; 57 (5): 419–433.

[4] Paaßen B, Morgenroth T, Stratemeyer M. What is a true gamer? The male gamer stereotype the marginalization of women in video game culture. Sex Roles. 2016; 76 (7): 421–435.

[5] Shen C, Ratan R, Dora Cai Y, Leavitt A. Do men advance faster than women? Debunking the gender performance gap in two massively multiplayer online games. J Comput Mediat Commun. 2016; 21: 312–329.

[6] Kuznekoff JH, Rose LM Communication in multiplayer gaming: Examining player responses to gender cues. New Media Soc. 2013; 15 (4): 541–556.

[7] Jenson J, de Castell S. Theorizing gender and digital gameplay: Oversights, accidents and surprises. Eludamos: J Comput Game Culture. 2008; 2(1): 15–25.

[8] Kafia YB, Hetter C, Denner J, Sun JY. *Beyond Barbie and Mortal Kombat*: *New perspectives on gender and gaming*. Cambridge, MA: MIT Press; 2008.

[9] Shapiro JR, Neuberg SL. From stereotype threat to stereotype threats: Implications of a multi-threat framework for causes, moderators, mediators, consequences and interventions. Pers Soc Psychol Rev. 2007; 11: 107–130. doi: 10.1177/1088868306294790 18453458 10.1177/1088868306294790

[10] Shapiro JR, Williams AM, Hambarchyan M. Are all interventions created equal? A multi-threat approach to tailoring stereotype threat interventions. J Pers Soc Psychol. 2013; 104(2): 277–288. doi: 10.1037/a0030461 23088232 10.1037/a0030461 PMC3682115

[11] Lewis A, Griffiths MD. Confronting gender representation: A qualitative study of the experiences and motivations of female casual-gamers. Aloma. 2011; 28: 245–272.

[12] Nguyen HD, Ryan AM. Does stereotype threat affect test performance of minorities and women? A meta-analysis of experimental evidence. J Appl Psychol. 2008; 93(6): 1314–1334. doi: 10.1037/a0012702 19025250 10.1037/a0012702

[13] Spencer SJ, Steele CM, Quinn DM. Stereotype threat and women’s math performance. J Exp Soc Psychol. 1999; 35: 4–28.

[14] Steele CM, Aronson J. Stereotype threat and the intellectual test performance of African Americans. J Pers Soc Psychol; 1995; 69(5): 797–811. 7473032 10.1037//0022-3514.69.5.797

[15] Stone J, Lynch CI, Sjomeling M, Darley JM. Stereotype threat effects on Black and white athletic performance. J Pers Soc Psychol. 1999; 77(6): 1213–1227.

[16] Pennington CR, Heim D. Creating a critical mass eliminates the effects of stereotype threat on women's mathematical performance. Br J Edu Psychol. 2016; 86 (3): 353–368. 10.1111/bjep.12110 27017194

[17] Doyle RA, Voyer D. Stereotype manipulation effects on math and spatial test performance: A meta-analysis. Learn Individ Differ. 2016; 43: 103–116.

[18] Pennington CR, Heim D, Levy A, Larkin D. Twenty years of stereotype threat research: A review of the psychological mediators. PLoS One. 2016; 11: 1–25. 10.1371/journal.pone.0146487 PMC4713435 26752551

[19] Flores PC, Wicherts JM. Does stereotype threat influence performance of girls in stereotyped domains? A meta-analysis. Journal of School Psychology. 2015; 53: 25–44. doi: 10.1016/j.jsp.2014.10.002 25636259 10.1016/j.jsp.2014.10.002

[20] Kaye LK, Pennington CR. “Girls can’t play”: The effects of stereotype threat on females’ gaming performance. Comput Human Behav. 2016; 59: 202–209.

[21] Vermeulen L, Castellar EN, Janssen D, Calvi L, Van Looy J. Playing under threat: Examining stereotype threat in female game players. Comput Human Behav. 2016; 57: 377–387.

[22] Shapiro JR. Different groups, different threats: A multi-threat approach to the experience of stereotype threats. Pers Soc Psychol Bull. 2011; 37(4): 464–480. doi: 10.1177/0146167211398140 21441217 10.1177/0146167211398140

[23] Wout D, Danso H, Jackson JS, Spencer S. The many faces of stereotype threat: Group- and self-threat. J Exp Soc Psychol. 2008; 44 (3): 792–799.

[24] Tajfel H. Differentiation between social groups London: Academic Press; 1978.

[25] Tajfel H. Individuals and groups in social psychology. Br J Soc Clin Psychol.1979; 18: 183–190.

[26] Tajfel H, Turner J. An integrative theory of inter-group conflict In Williams JA, Worchel S. (eds.) The social psychology of inter-group relations. Belmont, CA: Wadsworth; 1979 p. 33–47.

[27] Inzlicht M, Ben-Zev T. A threatening intellectual environment: Why females are susceptible to experiencing problem-solving deficits in the presence of males. Psychol Sci. 2000; 11 (5): 265–371. 10.1111/1467-9280.00272 11228906

[28] Inzlicht M, Ben-Zeev T. Do high-achieving female students underperform in private? The implications of threatening environments on intellectual processing. J Educ Psychol. 2003; 95: 796–805.

[29] Crocker J. Social stigma and self-esteem: Situation construction of self-worth. J Exp Soc Psychol. 1999; 35: 89–107.

[30] Blanton H, Crocker J, Miller DT. The effects of in-group versus out-group social comparison on self-esteem in the context of a negative stereotype. J Exp Soc Psychol. 2000; 36: 519–530.

[31] Desrichard O, Kӧpetz C. A threat in the elder: The impact of task-instructions, self-efficacy and performance expectations on memory performance in the elderly. Eur J Soc Psychol. 2005; 35(4): 537–552.

[32] Hoyt CL. The role of leadership efficacy and stereotype activation in women’s identification with leadership. J Leadership Organizational Stud. 2005; 11(4): 2–14.

[33] Milner HR, Hoy AW. A case study of an African American Teacher’s self-efficacy, stereotype threat and persistence. Teach Teach Educ. 2003; 19(2): 263–276.

[34] Soloman MA, Lee AM., Belcher D, Harrison L, Wells L. Beliefs about gender appropriateness, ability, and competence in physical activity. J Teach Phys Educ. 2003; 22: 261–279.

[35] Bogost I. *How to do things with videogames*. Minneqpolois: University of Minnesota Press; 2011.

[36] Shaw A. Do you identify as a gamer? Gender, race, sexuality and gamer identity. New Media Soc. 2012; 14 (1): 28–44.

[37] Vermeulen L, Van Bauwel S, Van Looy J. Tracing female gender identity: An empirical study into gender and stereotype threat perceptions. Comput Human Behav. 2017; 71: 90–98.

[38] Steele CM, Davies PG. Stereotype threat and employment testing: A commentary. Hum Perform, 2003; 16 (3): 311–326.

[39] Schmader J. Gender identification moderates stereotype threat on women’s math performance. J Exp Soc Psychol. 2002;38: 194–201.

[40] Cameron J. A three factor model of social identity. Self Identity. 2004; 3 (3), 239–262.

[41] Obst PL, White KM Three-dimensional strength of identification across group memberships: A confirmatory factor analysis. Self Identity. 2005; 4: 69–80.

[42] Rosenberg M. *Society and the Adolescent Self-Image*. Princeton, NJ: Princeton University Press; 1965.

[43] Schwarzer R, Jerusalem M. Generalized self-efficacy scale. In Weinman J, Wright S, Johnston M.(eds.) Measures in health psychology: A user’s portfolio. Causal and control beliefs. Windsor, UK; 1995 p. 35–37.

[44] Cohen J. A power primer. Psychol Bull. 1992; 112: 155–159. 19565683 10.1037//0033-2909.112.1.155

[45] Hayes AF. *Introduction to Mediation*, *Moderation and Conditional Process Analysis*: *A Regression-based Approach*. New York: Guilford Press; 2013.

[46] Vermeulen L, Van Looy J. “I Play So I Am?” A Gender Study into Stereotype Perception and Genre Choice of Digital Game Players. J Broadcast Electron Media. 2016; 60 (2): 286–304.

[47] Ganley CM, Mingle LA, Ryan AM, Ryan K, Perry M. An examination of stereotype threat effects on girls’ mathematics performance. DevPsychol. 2013; 49: 1886–1897. 10.1037/a0031412 23356523

[48] Schmader T, Johns M Converging evidence that stereotype threat reduces working memory capacity. J Pers Soc Psychol. 2003;85(3): 440–452. doi: 10.1037/0022-3514.85.3.440 14498781 10.1037/0022-3514.85.3.440

[49] Schmader T, Johns M, Forbes C. An integrated process model of stereotype threat effects on performance. Psychol Rev. 2008;115: 336–356. doi: 10.1037/0033-295X.115.2.336 18426293 10.1037/0033-295X.115.2.336 PMC2570773

[50] Molden DC. Understanding priming effects in social psychology” what is “social priming” and how does it occur? Soc Cogn. 2014; 32: 1–11.

[51] Lee JER, Nass C. Distinctiveness-based stereotype threat and the moderating role of coaction contexts. J Exp Soc Psychol. 2012; 48 (1): 192–199.

[52] De Grove F, Courtois C, Van Looy J. How to be a gamer! Exploring personal and social indicators of gamer identity. J Comput Mediat Commun. 2015; 20(3): 346–361.

[53] Schwarz N. Self-reports: How the questions shape the answers. Am. Psychol. 1999; 54 (2): 93

